# Structural Stigma, Racism, and Sexism Studies on Substance Use and Mental Health: A Review of Measures and Designs

**DOI:** 10.35946/arcr.v44.1.08

**Published:** 2024-12-18

**Authors:** James K. Cunningham, Ahlam A. Saleh

**Affiliations:** 1 Department of Family and Community Medicine, University of Arizona, Tucson, Arizona; 2 Health Sciences Library, University of Arizona, Tucson, Arizona

**Keywords:** alcohol, structural stigma, structural racism, institutional racism, structural sexism, structural determinants of health, substance use, mental health

## Abstract

**PURPOSE:**

Most research on the structural determinants of substance use and mental health has centered around widely studied factors such as alcohol taxes, tobacco control policies, essential/precursor chemical regulations, neighborhood/city characteristics, and immigration policies. Other structural determinants exist, however, many of which are being identified in the emerging fields of structural stigma, structural racism, and structural sexism. This narrative review surveys the measures and designs used in substance use and mental health studies from these three fields.

**SEARCH METHODS:**

The PubMed, PsycINFO, and Scopus databases were searched on May 11, 2023. A focused search approach used terminology for structural racism, stigma, or sexism combined with terminology for substance use or mental health. Peer-reviewed studies were included if they were written in English and assessed associations between objective structural measures and substance use and mental health outcomes.

**SEARCH RESULTS:**

Of 2,536 studies identified, 2,487 were excluded. Forty-nine studies (30 related to stigma, 16 related to racism, and three related to sexism) met the inclusion criteria. Information was abstracted about the structural measures, outcome measures, research design, sample, and findings of each study.

**DISCUSSION AND CONCLUSIONS:**

The structural determinant measures used in the studies reviewed were diverse. They addressed, for example, community opinions, the gender of legislators, economic vulnerability, financial loan discrimination, college policies, law enforcement, historical trauma, and legislative protections for sexual and gender minorities and for reproductive rights. Most of the structural determinant measures were constructed by combining multiple indicators into indexes or by merging indexes into composite indexes, although some studies relied on single indicators alone. The substance use and mental health outcome measures most frequently examined were related to alcohol and depression, respectively. The studies were conducted in numerous nations and drew samples from an array of groups, including, for example, patients who experienced overdoses from substance use, sexual and gender minorities, racial and ethnic minority groups, women, youth, migrants, and patients subject to involuntary psychiatric hospitalization. Most of the studies used passive-observational (correlational) research designs and, as a result, did not assess whether their structural determinant variables were causally related to substance use and mental health. Nevertheless, the studies reviewed can be used by public health proponents to foster awareness that a wide range of structural determinants correlate with the substance use and mental health of many groups within and across nations.

Factors such as government policies, institutional practices, economic systems, social norms, and the physical environment can impact health. Called structural determinants of health in medical parlance,^1^ several of these factors known to be associated with substance use and mental health have long been studied. Examples include alcohol taxation (associated with alcohol use and mortality),^2^–^4^ alcohol outlet density (associated with alcohol-related health),^5^ tobacco taxes (associated with tobacco use),^6^ tobacco control policies (associated with smokeless tobacco use),^7^ essential/precursor chemical regulations (associated with illicit drug use and morbidity),^8^–^11^ neighborhood/city characteristics (associated with mental health),^12^,^13^ and immigration policies (associated with mental health).^14^,^15^

However, other structural determinants of substance use and mental health exist, many of which are being identified in the emerging fields of structural stigma, structural racism, and structural sexism. Although the need to consider measures from these fields has been discussed,^16^–^21^ a survey of the measures and related research elements used in substance use and mental health studies across the three fields has not been conducted to date. This narrative review helps address this gap.

## Definitions of Structural Stigma, Structural Racism, and Structural Sexism

As background, it will be helpful first to outline what structural stigma, structural racism, and structural sexism are. Hatzenbuehler and Link defined structural stigma as the “societal-level conditions, cultural norms, and institutional policies that constrain the opportunities, resources, and well-being of the stigmatized.”^22^^(p2)^ Examples of stigmatized groups are persons living with human immunodeficiency virus (HIV), persons living with mental illness, persons who misuse substances, sexual and gender minorities, and marginalized racial and ethnic groups.^19^

According to Bailey et al., structural racism involves interconnected institutions, whose linkages are historically rooted and culturally reinforced: “It refers to the totality of ways in which societies foster racial discrimination, through mutually reinforcing inequitable systems (in housing, education, employment, earnings, benefits, credit, media, health care, criminal justice, and so on) that in turn reinforce discriminatory beliefs, values, and distribution of resources, which together affect the risk of adverse health outcomes.”^23^^(p1454)^ Academics often use the terms “structural racism” and “institutional racism” interchangeably.^17^,^24^ Institutional racism, as defined by Willams et al., “refers to the processes of racism that are embedded in laws (local, state, and federal), policies, and practices of society and its institutions that provide advantages to racial groups deemed as superior, while differentially oppressing, disadvantaging, or otherwise neglecting racial groups viewed as inferior.”^24^^(p107)^Javidan defined structural sexism as “discriminatory beliefs or practices on the basis of sex and gender that are entrenched in societal frameworks and which result in fairly predictable disparities in social outcomes related to power, resources, and opportunities. Sexism is structural when it is integral to how a society is organized, which can be observed in its institutions, systems, and/or patterns of social relations. Structural sexism also functions to normalize and legitimize such beliefs, practices, and inequalities of conditions and outcomes.”^25^^(p1)^

## Approach of the Review

This narrative review surveys the objective measures of structural determinants (e.g., government policies, institutional actions, community opinions/norms), the substance use and mental health outcome measures, and the research designs used in studies of structural stigma, structural racism, and structural sexism. Objective measures of structural determinants are considered here for multiple reasons. Compared with self-reports of structural determinants from individuals (e.g., self-reports of discrimination at a workplace), objective structural measures avoid issues such as respondents’ bias or lack of awareness of structural effects.^16^ Objective structural measures also avoid same-source bias, which can introduce spurious results when the independent and dependent variables are measured with the same method.^19^,^26^ Finally, as noted by Groos et al., objective quantitative measurement is important to the identification of reliable and causal associations between structural determinants and health.^18^

## Methods

### Search Strategy

The PubMed, PsycINFO, and Scopus databases were searched for articles on May 11, 2023, with the help of a health sciences librarian and using a focused search approach. Searches included thesaurus terms, such as Medical Subject Headings (MeSH), when available, as well as text words to search in the title or abstract (tiab), or author-supplied keyword fields of the article records in the databases. The terms “structural,” “institutional,” “institutionalized,” or “systemic” were paired with the terms “stigma,” “racism,” “barriers,” and “sexism”; all of these were combined with terms for substance use or mental health.

The following search strategy for PubMed demonstrates the search terms used and the actual search strategy that was employed and then translated to the other databases: ((“Systemic Racism” [MeSH] OR “systemic racism” [tiab: ~3] OR “systemic stigma” [tiab: ~3] OR “systemic barriers” [tiab: ~3] OR “systemic sexism” [tiab: ~3] OR “structural racism” [tiab: ~3] OR “structural stigma” [tiab: ~3] OR “structural barriers” [tiab: ~3] OR “structural sexism” [tiab: ~3] OR “stigma system” [tiab: ~3] OR “racism system” [tiab: ~3] OR “institutional racism” [tiab: ~3] OR “institutional stigma” [tiab: ~3] OR “institutional barriers” [tiab: ~3] OR “institutional sexism” [tiab: ~3] OR “institutionalized racism” [tiab: ~3] OR “institutionalized stigma” [tiab: ~3] OR “institutionalized barriers” [tiab: ~3] OR “institutionalized sexism” [tiab: ~3]) AND (“Substance-Related Disorders” [MeSH] OR “Drug Misuse” [MeSH] OR Smoking [MeSH] OR “Substance Abuse Treatment Centers” [MeSH] OR “Mental Disorders” [MeSH] OR “Mental Health” [MeSH] OR ““Mental Health Services” [MeSH] OR “Community Mental Health Centers” [MeSH] OR “substance use” [tiab] OR “substance abuse” [tiab] OR “substance dependence” [tiab] OR “substance misuse” [tiab] OR “drug use” [tiab] OR “drug abuse” [tiab] OR “drug misuse” [tiab] OR “use disorder*” [tiab] OR “addiction” [tiab] OR “mental health” [tiab] OR “mental disorder*” [tiab] OR “mental illness” [tiab])).

The MeSH terms listed above often had descendent MeSH terms that were automatically searched. For example, the MeSH term “Mental Disorders” had descendent MeSH terms such as “Anxiety Disorders,” “Mood Disorders,” and “Schizophrenia Spectrum and Other Psychotic Disorders”; many of these had their own descendent MeSH terms, all of which were automatically searched.

### Eligibility Criteria

Article inclusion criteria were as follows: (1) original research, (2) peer-reviewed, (3) written in English, and (4) one or more objective structural determinant measures used to help explain behavioral health outcomes (e.g., self-reports from individuals regarding alcohol use or depression). Other types of studies on structural determinants (e.g., commentaries, review papers, qualitative analyses, studies with no behavioral health outcome measures) were excluded.

### Literature Search Results

The PubMed, PsycINFO, and Scopus searches identified 2,020, 1,639, and 827 records, respectively. Duplicates (*n* = 1,950) were eliminated, leaving a total of 2,536 records to review. The titles and abstracts were reviewed for relevance to the topic, and the full text of the resulting selected (potentially relevant) studies were reviewed for further analysis. This resulted in the exclusion of 2,487 studies. Forty-nine studies (30 related to structural stigma,^27^–^56^ 16 related to structural racism,^57^–^72^ and three related to structural sexism^73^–^75^) met the inclusion criteria and are reviewed here.

## Results

The number of structural stigma, racism, and sexism studies examining substance use and mental health has recently accelerated. The first identified study was published in 2004.^30^ Eight were published during 2004 through 2017, eight were published during 2018 through 2020, and 33 were published during 2021 through May 11, 2023; this last period included 11 studies on substance use and 22 studies on mental health.

Thirty-six of the 49 studies identified were conducted in the United States; this included 18 of 30 structural stigma studies, 15 of 16 structural racism studies, and all three of the structural sexism studies. Six of the 12 structural stigma studies conducted outside the United States were multinational in that they examined samples drawn from a range of four countries to more than 100 countries;^30^,^41^,^43^,^45^,^51^,^54^ the remaining structural stigma studies were from Australia,^47^ Brazil,^46^ Italy,^50^ Sweden,^36^, ^55^ and the United Kingdom.^34^ The one structural racism study conducted outside of the United States was from England.^57^

### Outcome Measures

Fifteen studies examined the association between structural determinants and substance use-related outcome measures. Approximately twice that number (31 studies) examined the association between structural determinants and mental health-related outcome measures. Another three studies examined how structural determinant measures were associated with both substance use- and mental health-related outcomes. All studies are summarized in Appendix 1.

Measures related to alcohol use constituted the substance use outcome category most often examined (Table 1). However, multiple studies also examined tobacco use, other drug use, and/or substance use-related services. When different studies considered the same type of substance use outcome (e.g., binge drinking), there was often variation in the specific measures used. For example, Greene et al.^35^ defined binge drinking as four or more drinks for women and five or more drinks for men on a single occasion in the past 30 days; Drabble et al. defined it as eight or more drinks in a single day during the past year;^31^ Everett et al. defined it as six or more drinks in a day in the past 12 months;^32^ and McKetta et al. (in two studies) defined it as five or more drinks in a row during the past 2 weeks.^74^, ^75^ Among the four studies that had smoking outcome measures, one considered cigarette use in the past year,^37^ one examined current use of cigarettes,^53^ one examined 7-day abstinence from cigarettes at week 26 of a clinical trial,^68^ and one examined current smoking at the census tract level without specifying whether it was limited to cigarettes.^66^

The mental health outcome most often examined was depression (Table 2). Multiple studies also considered anxiety, eating disorders, general mental health, and mental health-related services. Several studies used the same mental health outcome measures. For example, six studies assessed depression using versions of the Center for Epidemiologic Studies-Depression (CES-D) scale,^32^,^39^,^41^,^48^,^50^,^72^ and five assessed anxiety using the General Anxiety Disorder-7 (GAD-7) scale.^34^,^39^,^45^,^56^,^67^

Nearly all of the substance use-related studies (17 of 18) were conducted in the United States (see Appendix 1). In contrast, 12 of the 34 studies examining mental health-related outcome measures were conducted outside of the United States. (Note that the three studies^27^,^32^,^66^ that examined both substance use- and mental health-related outcomes were included in the above count of 18 substance use studies and again in the count of 34 mental health studies.)

### Structural Determinant Measures

Government legislation/policy was the subject area most often addressed by structural determinant measures (it was used in 23 studies); examples include legislative protections for sexual and gender minorities and for reproductive rights, legislative bills with stigmatizing language, and the gender and political parties of legislators (see Appendix 1). However, government legislation/policy was not used as a structural determinant in any of the structural racism studies. The other three top structural determinants measured were community opinions/norms (e.g., neighborhood racism, community opinions regarding sexual minorities), which were used in 15 studies; area characteristics (e.g., areas with low socioeconomic status, housing remediation, segregation), which were used in 13 studies; and institutional policy/action (e.g., financial loan discrimination, college policies to promote inclusion of sexual and gender minorities), which were used in eight studies. Various other determinants (e.g., regional development, law enforcement, historical trauma) were examined as well.

No two different lead authors used the exact same measures for structural determinants. That said, several studies constructed structural determinant measures using data from the same sources. For example, nine studies used U.S. Census data;^41^,^59^,^62^,^64^–^68^,^70^ three used data from the Movement Advancement Project;^27^,^31^,^42^ and three used data (grades) from the Home Owners’ Loan Corporation.^64^,^66^,^67^

The studies’ structural determinant measures can be classified into four types: single indicators, multiple single indicators, indexes, and composite indexes. An example of a single indicator would be a community opinion/norm or the enactment of new legislation. An example of multiple single indicators would be the use of a community opinion/norm and new legislation as individual structural determinant measures in the same study. Indexes consist of multiple related indicators (e.g., two different financial lending measures) combined into one measure. A composite index is a single measure constructed by combining an indicator/index for one topic with an indicator/index for at least one other topic. An example would be the combining of an index for a state’s policies with an indicator of poverty in that same state.

The most frequently used type of structural determinant measure was the index (used in 17 studies). This was followed by composite indexes (13 studies), single indicators (13 studies), and multiple single indicators (six studies). All four types were used in structural stigma studies (seven used single indicators,^32^,^34^,^39^,^40^,^46^,^47^,^50^ four used multiple indicators,^29^,^33^,^41^,^56^ nine used indexes,^27^,^28^,^31^,^35^,^42^,^48^,^49^,^51^,^52^ and 10 used composite indexes^30^,^36^–^38^,^43^–^45^,^53^–^55^). None of the structural racism studies used composite indexes; eight used indexes,^59^,^61^,^62^,^64^,^65^,^67^,^68^,^70^ six used single indicators,^57^,^58^,^63^,^66^,^71^,^72^ and two used multiple single indicators.^60^, ^69^ All three of the structural sexism studies used composite indexes.^73^–^75^

Compared to composite indexes, the use of indexes might be on the rise. Sixteen of the 17 studies using indexes were published in 2021 or later, whereas only eight of the 13 studies using composite indexes were published in 2021 or later.

### Types of Research Designs Used

None of the 49 studies reviewed used experimental designs. Seven used quasi-experimental designs/methods: Specifically, three used pretest-posttest comparisons (two with pretest groups that differed from their posttest groups and none with a comparison group),^32^,^39^,^50^ two used propensity score matching,^42^, ^73^ one used a difference-in-differences design,^71^ and one used an interrupted time series design.^40^ These latter two designs were among the strongest regarding causal inference, but the studies using them did not find statistically significant associations between their structural determinant measures and outcome measures. Forty-two of the 49 studies used passive-observational designs (i.e., correlational designs including, for example, regressions with control variables, path analyses, and structural equation models). Five of these examined ecological correlations (i.e., correlations in which the units studied were populations or groups rather than individuals).^33^,^52^,^58^,^64^,^66^ Whether studies examined structural stigma, structural racism, or structural sexism, passive-observational designs predominated.

### Types of Associations

Most of the studies (39 of 49) focused exclusively on direct associations between their structural determinant and outcome measures. Five studies (one on substance use^74^ and four on mental health^36^,^43^,^62^,^65^) considered both direct and indirect associations. Five studies (all mental health studies) reported indirect or moderator associations but not direct associations between their structural and outcome measures.^34^,^54^–^56^,^61^

The majority of the studies reported that statistically significant associations were found between their structural measures and outcome measures. Whether the associations found had practical significance was generally not discussed.

### Groups Sampled

Sexual minority groups were sampled more often than any other group (Table 3). Racial and ethnic minority groups, primarily persons who were Black, were sampled second most often. Other groups sampled included, for example, migrants, youth, women, psychiatric patients subject to involuntary hospitalization, and patients who experienced overdoses from substance use. Sometimes the samples consisted of items rather than people. For instance, Conley^28^ and Conley and Baum^29^ analyzed samples of mental health-related bills from all 50 states.

The three fields tended to differ regarding groups examined. For example, almost all of the studies that considered sexual and gender minority groups were structural stigma studies. Most of the studies that focused on persons who were Black were structural racism studies. All three of the structural sexism studies used surveys that primarily sampled cisgender persons.

## Discussion

This narrative review found a substantial, recent rise in substance use studies and mental health studies within the collective fields of structural stigma, structural racism, and structural sexism. Although increases in both types of studies contributed to the overall rise, those involving mental health exceeded those involving substance use by about two to one.

The outcome measures used in the studies (e.g., measures of alcohol use and depression) were generally the same or similar to those commonly used in substance use and mental health epidemiology analyses. Notwithstanding this, the structural determinant measures used were diverse and often innovative, at least in comparison to those used in the long-standing lines of structural determinant research (e.g., alcohol taxation, essential/precursor chemical control, immigration policy) noted earlier. The structural determinant measures of the reviewed studies addressed, for example, community opinions about social issues, the gender of legislators, financial loan discrimination, economic vulnerability, college policies, law enforcement practices, historical trauma, and legislative protections for reproductive rights and for sexual and gender minorities.

The structural measures were also relatively novel in that more than half were indexes or composite indexes. This differs from the structural determinant measures used in alcohol taxation and other long-standing lines of structural determinant research, which typically have used single indicators. Some of the indexes and composite indexes in the reviewed studies were complex. For example, Beccia et al. used a composite index that assessed political participation, state policies, socioeconomic measures, institutional support, and access to reproductive care.^73^

In the studies reviewed, the use of indexes for measuring structural determinants increased over time. Authors have debated the use of single indicators versus indexes and composite indexes.^76^, ^77^ Single indicators may provide clearer guidance for policy in the sense that each indicator’s contribution to an outcome can be described. On the other hand, an index or composite index provides a single score representing a set of structural determinants, and policymakers and the public may be more comfortable considering that single score as opposed to pondering how each of several single indicators contributes to an outcome. If the goal of a study is to guide policy directly, single indicators may be preferable. If the goal is to increase awareness of structural determinants, then indexes or composite indexes may be more helpful.

When examining multiple structural determinants, future studies could consider performing one analysis with single indicators and another using indexes or composite indexes. The former could help provide direct guidance regarding individual determinants, whereas the latter could provide a simpler—and perhaps easier to remember—representation of the structural determinants’ overall impact.

Little information on whether structural determinants and behavioral health outcomes were causally related was provided, as most of the reviewed studies used passive-observational (correlational) research designs. Seven used quasi-experimental designs. Of these, the two studies that arguably had the strongest designs—a difference-in-differences design and an interrupted time series design—reported null effects.^40^, ^71^ Relatively strong quasi-experimental designs are usually needed to help evaluate whether structural determinants are responsible for changes in behavioral health outcomes.^78^–^80^ Such designs have commonly been used in some of the long-standing lines of structural determinant-behavioral health research; this includes studies on alcohol taxation,^2^–^4^ tobacco taxation,^6^ and essential/precursor chemical regulation.^8^–^11^

Although most of the reviewed studies reported statistically significant results, they generally did not discuss practical significance.^81^ These two types of significance can differ as illustrated in the following example. In a trial of a vaccine for the common cold, people who received the vaccine had an average of 1.6 colds per year, whereas those who did not receive the vaccine averaged 2.1 colds. The difference in the number of colds between the two groups had high statistical significance (p < .000001). Nevertheless, the difference was thought to lack practical significance because an average of 0.5 fewer colds per year was considered too small to justify the vaccine’s expense and possible side effects.^82^, ^83^ Similarly, when examining structural determinants, it is usually important to consider not only the statistical significance of an effect (association) but also its practical significance. For example, is the effect substantial enough to warrant real-world actions such as policy changes, efforts to shift social norms, or the introduction of new legislation?

The studies reviewed drew samples from a varied array of groups (see Appendix 1 and Table 3). However, within each of the three fields, some groups tended to be sampled with particular frequency. Structural stigma studies, for example, often considered sexual and gender minority persons, structural racism studies often considered persons who were Black, and structural sexism studies analyzed survey samples predominantly composed of cisgender individuals. Numerous groups remain to be examined. For instance, although 18 studies considered outcomes related to substance use, none were specific to persons who used methamphetamine, cocaine, or psychedelics. None of the studies focused on persons who were homeless or who had physical, intellectual, or psychiatric disabilities.^84^ Also, none of the studies analyzed population samples specific to American Indians, Alaska Natives, Hawaiian Natives, or other Indigenous people in colonized countries.

Interest in the study of structural stigma, structural racism, and structural sexism appeared to differ geographically. Structural stigma studies were conducted in a wide range of nations, while all of the structural sexism studies and all but one of the structural racism studies were conducted in the United States.

### Limitations

This review was exclusive to structural stigma, structural racism, and structural sexism studies that used objective structural determinant measures to help explain outcomes related to substance use and mental health. Studies from these fields that used qualitative analyses and self-reports to assess structural determinants related to substance use and mental health were not considered and remain to be reviewed. Bias may have occurred due to not including manuscripts from the gray literature. This review focused on measures and designs, not on study findings. Nations can differ regarding policies, norms, economic systems, and so on. Such differences could limit the extent to which associations between structural determinants and behavioral health outcomes can be generalized from one nation to another.

### Conclusions

Structural stigma, structural racism, and structural sexism studies using objective structural determinant measures to help explain behavioral health issues have recently accelerated in number. These studies employed a diverse array of structural determinant measures, many of which were innovative. However, most of the studies used passive-observational (correlational) research designs and thus provided little evidence as to whether causal associations existed between the structural determinants and the substance use and mental health outcomes examined. Moving forward, relatively strong quasi-experimental designs could be used to help address this limitation. In the meantime, public health proponents can use the studies reviewed to help foster awareness that a wide range of structural determinant measures correlate with the substance use and mental health of many groups within and across nations.

**KEY TAKEAWAYS**
- Studies on substance use and mental health issues related to structural stigma, racism, and sexism recently have increased sharply in number.
- The structural determinant measures used in the studies were diverse and often innovative.
- Most of the studies used passive-observational (correlational) designs, which limited the ability to assess whether the structural determinants examined were causally related to substance use and mental health.
- The studies typically did not discuss the practical significance of the statistically significant associations found between structural determinants and substance use and mental health.
- Collectively, the studies showed that a wide range of structural determinants correlate with the substance use and mental health of many groups within and across nations.

## Figures and Tables

**Table 1:** Substance Use Outcome Categories: Number of Structural Stigma, Structural Racism, and Structural Sexism Studies

Outcome	Total Studies	Structural Stigma Studies	Structural Racism Studies	Structural Sexism Studies
Alcohol*	10	6 (31,32,35,40,44,51)	2 (66,71)	2(74,75)
Tobacco Use	5	3 (37,44,53)	2 (66,68)	0
Marijuana Use	2	2 (31,38)	0	0
Illicit Drug Use	3	2 (38,51)	1 (71)	0
Substance Use-Related Services	3	3 (33,40,52)	0	0
Drug-Related Deaths	1	0	1 (70)	0

*Note:* Reference numbers are in parentheses. Studies that examined multiple outcome categories are counted in each of those categories.

* Any alcohol-related outcome (does not include substance use-related services)

**Table 2:** Mental Health Outcome Categories: Number of Structural Stigma, Structural Racism, and Structural Sexism Studies

Outcome	Total Studies	Structural Stigma Studies	Structural Racism Studies	Structural Sexism Studies
Depression	13	7 (32,39,41,43,48,50,54)	6 (58,61,65,67,69,72)	0
General Mental Health	8	5 (28,29,42,47,55)	3 (60,64,66)	0
Anxiety	7	5 (34,39,45,50,56)	2 (61,67)	0
Stress/Worry	4	2 (35,56)	2 (59,63)	0
Eating Disorders	2	0	1 (62)	1 (73)
Mental Health-Related Services	6	4 (27,30,46,49)	2 (57,58)	0

*Note:* Reference numbers are in parentheses. Studies that examined multiple outcome categories are counted in each of those categories.

**Table 3:** Groups Sampled: Number of Structural Stigma, Structural Racism, and Structural Sexism Studies

Groups Sampled*	Total Studies	Structural Stigma Studies	Structural Racism Studies	Structural Sexism Studies
**Sexual and Gender Groups**				
Sexual Minority^†^	17	16 (31,32,35–38,41–45,47,49,50,53,55)	1 (60)	0
Gender Minority	1	1 (40)	0	0
Sexual/Gender Minorities	4	4 (27,39,54,56)	0	0
Females	2	0	0	2 (74,75)
**Racial and Ethnic Groups**				
Black Individuals	10	0	10 (58,59,61,62,65, 67–69,71,72)	0
Latino Individuals	1	0	1 (63)	0
Black and Latino Individuals	1	0	1 (70)	0
**Other Groups of Persons**				
General Population	3	1 (51)	2 (64,66)	0
People who Inject Drugs	1	1 (33)	0	0
Migrants	1	1 (34)	0	0
Patients Experiencing Overdoses	1	1 (52)	0	0
Patients in Mental Health Hospitals	1	0	1 (57)	0
Persons With Behavioral Disorders	1	1 (30)	0	0
Youth	3	2 (46,48)	0	1 (73)
**Items/Entities**				
Mental Health-Related Legislative Bills	2	2 (28,29)	0	0

*Note:* Reference numbers are in parentheses.

* Groups were categorized according to the major focus of a study. For instance, in the Miedema et al. study of sexual minority men in four Asia-Pacific region countries,^41^ the focus was primarily on sexual minority status. The study consequently was listed in the sexual minority category.

Includes one study that sampled sexual minority youth of color.

**Appendix 1:** Studies on the Association of Substance Use and Mental Health With Structural Stigma, Structural Racism, and Structural Sexism

Author (Year)Nation	Type and Subject of Structural Measure*	Structural Measure	Sample	Outcome Measure	Finding	Field Design
**Substance Use Studies**						
**Outcome: Alcohol Use**						
Greene et al. (2021)^35^ United States	Index—government legislation/policy	Alcohol Policy Scale, an index that measures the presence and implementation of alcohol policies in a state. Binary indicator of whether a state’s nondiscrimination laws explicitly include sexual orientation (i.e.,inclusive nondiscrimination statutes).	LGB and heterosexual adults from the Behavioral Risk Factor Surveillance System (BRFSS)	Binge drinking: 4+ drinks for females and 5+ drinks for males on a single occasion in the past 30days	Higher scores on the Alcohol Policy Scale were associated with less binge drinking among women (but not men) in states with inclusive nondiscrimination statutes. Binge drinking disparities between lesbian/bisexual women and heterosexual women were narrower in states with inclusive nondiscrimination statutes.	Structural stigma Passive observational
McKetta et al. (2022)^74^ United States	Composite index—government legislation/policy with other subjects	Using factor analysis, a scalar score for structural sexism at the state level was constructed. It was based on the percentage of male state legislators and on the state’s male/female ratio for the proportion of (1) residents living at or above the federal poverty line, (2) adults age 16 and older in the labor force, (3) adults in management occupations, and (4) working adults who are self-employed. The scalar score varied with time.	Female respondents (ages 27 to 45 in 2016) to the Monitoring the Future survey (*N =* 20,859) from 1988 to 2016	Occasions in the past month that respondents had 1+ drinks. Binge drinking (5+ drinks in a row during past 2 weeks)	Lower levels of state structural sexism were directly and indirectly associated with greater alcohol consumption and binge drinking.	Structural sexism Passive observational
McKetta et al. (2023)^75^ United States	Composite index—government legislation/policy with other subjects	Using factor analysis, a scalar score for structural sexism at the state level was constructed. It was based on the percentage of male state legislators and on the state’s male/female ratio for the proportion of (1) residents living at or above the federal poverty line, (2) adults age 16 and older in the labor force, (3) adults in management occupations, and (4) working adults who are self-employed. The scalar score varied with time.	Female respondents (ages 19 to 45) to the Monitoring the Future survey (*N =* 16,571) from 1989 to 2016	Occasions in the past month that respondents had 1+ drinks. Binge drinking (5+ drinks in a row during past 2 weeks)	In states with lower structural sexism, working and having a high-status career were associated with a greater number of drinking occasions.	Structural sexism Passive observational
**Outcome: Alcohol and Other Drug Use**						
Drabble et al. (2021)^31^ United States	Index—government legislation/policy	State policy environment was assessed using a time-varying dichotomous indicator of comprehensive protections for sexual minorities (4 to 5 protections vs. limited or no protections). Data on the policy environment were obtained from the Movement Advancement Project.	Respondents, including sexual minority men and women, to the National Alcohol Survey (*N =* 29,571)	Alcohol use disorder, high-intensity drinking (8+ drinks in a single day during past year) and marijuana use during past year	Higher scores on the policy index were associated with lower odds of high-intensity drinking among sexual minority men.	Structural stigma Passive observational
Pachankis et al. (2014)^44^ United States	Composite index—government legislation/policy with other subjects	Within states, the following policies were counted: (1) constitutional amendments banning same-sex marriage, (2) sexual orientation employment nondiscrimination laws, (3) statutes recognizing sexual orientation employment nondiscrimination laws, (4) nondiscrimination laws extending to students and/or a statute banning bullying based explicitly on sexual orientation, and (5) statutes that restrict same-sex couples from adopting or that make it difficult for non-married couples to adopt. By state, mean scores were constructed using polls about gay adoption, hate crimes, health benefits, discrimination in jobs and housing, marriage, sodomy, and civil unions (the mean scores were obtained from other researchers). These two measures were combined into a composite index.	Sexual minority persons who completed the study’s online survey (*N =* 119)	Any use of alcohol or tobacco on each of 9 consecutive days.	Current exposure to structural stigma was associated with increased tobacco use, but was not found to be associated with alcohol use.	Structural stigma Passive observational
**Outcome: Tobacco Use**						
Hatzenbuehler et al. (2014)^37^ United States	Composite index—government legislation/policy with other subjects	Using factor analysis, a composite index was constructed using four variables: (1) density of same-sex partner households by state, (2) proportion of Gay-Straight Alliances at high schools within each state, (3) index of state-level policies protecting LGB individuals, and (4) public opinion toward sexual minorities in each state.	Sexual minority and heterosexual adolescents participating in the Growing Up Today Study	Smoked cigarettes in the past year	No statistically significant association was found between the composite index and cigarette smoking.	Structural stigma Passive observational
Titus et al. (2021)^53^ United States	Composite index—government legislation/policy with other subjects	Composite index consisting of three primary components: (1) state-level policies related to sexual minorities, (2) state-level density of same-sex couple households, and (3) public opinion toward same-sex marriage	3,174 sexual minority adults and 105,803 heterosexual adults responding to the National Adult Tobacco Survey	Currently smoke cigarettes	The structural stigma index had an inverse curvilinear association with smoking. The association was found for both sexual minority and heterosexual respondents, but was more pronounced for the former.	Structural stigma Passive observational
Nollen et al. (2022)^68^ United States	Index—area characteristics	Indicators of area-level disadvantage derived from U.S. census tract 5-year estimates were linked to study participants’ home addresses. Census tract-level disadvantage was measured using percentage of female-headed households, public assistance, unemployed, < 100% of the federal poverty level, and whether > 25% had less than a high school education. A neighborhood disadvantage index score was created by calculating the z scores for each variable and summing them.	223 Black and 221 White low-income participants in a smoking cessation trial	Abstinence from cigarettes for 7 days at week 26 of the trial	Abstinence was less for persons in areas with greater disadvantage.	Structural racism Passive observational
**Outcome: Substance Use Services**						
Friedman et al. (2007)^33^ United States	Multiple single indicators—government legislation/policy	Within metropolitan statistical areas (MSAs), education expenditures per capita, percentage of drug users in treatment who are non-injection drug users, per capita long-term debt of governments in the MSA, percentage of population that is non-Hispanic White, and presence of organizations that support treatment	94 MSAs with populations greater than 500,000	Within MSAs, proportion of injection drug users in treatment	Presence of organizations that support treatment, higher education expenditures per capita, higher percentage of population that is non-Hispanic White, lower percentage of non-injection drug users in treatment, and lower long-term debt of governments were associated with more injection drug users in treatment.	Structural stigma Passive observational (ecological correlation)
Stokes et al. (2023)^52^ United States	Index—social vulnerability	Counties were ranked using the Centers for Disease Control and Prevention’s Social Vulnerability Index.	Emergency department visits and hospitalizations in selected counties	Within counties, nonfatal drug overdose emergency department visits and hospitalization rates	Higher vulnerability scores were associated with higher nonfatal drug overdose emergency department visit and hospitalization rates.	Structural stigma Passive observational (ecological correlation)
Matson et al. (2022)^40^ United States	Single indicator—institutional policy/action	Veterans Health Administration (VHA) Transgender Healthcare Directive—a national policy to reduce structural discrimination on receipt of evidence-based alcohol-related care among transgender patients with unhealthy alcohol use	VHA transgender patients with unhealthy alcohol use	Receipt of brief intervention for alcohol use, receipt of pharmacotherapy, receipt of special addictions therapy, and receipt of any of the preceding services	Using an interrupted time-series analysis, the directive was not found to be associated with an increase in any of the care measures related to substance use.	Structural stigma Quasi-experimental
**Outcome: Substance Use—Other**						
Hatzenbuehler et al. (2015)^38^ United States	Composite index—government legislation/policy with other subjects	Using factor analysis, a composite index was constructed using four variables: (1) density of same-sex partner households by state, (2) proportion of Gay-Straight Alliances at high schools within each state, (3) index of state-level policies protecting LGB individuals, and (4) public opinion toward sexual minorities in each state.	Sexual minority and heterosexual adolescents participating in the Growing Up Today Study	Self-reports of marijuana and illicit drug use	Risk ratios indicated that the difference in marijuana use between sexual minorities and heterosexuals was greater in high- vs. low-structural stigma states, and that, for females only, the difference in illicit drug use between sexual minorities and heterosexuals was also greater in high- vs. low-structural stigma states.	Structural stigma Passive observational
Simha et al. (2022)^51^ Multinational	Index—community opinions/norms	Measures of institutional collectivism, assertiveness, future orientation, and power distance dimensions at the country level obtained from the Global Leadership and Organizational Behavior Effectiveness (GLOBE) study	68,041 respondents from 49 countries	Respondents indicated their desire not to have a neighbor who consumed alcohol or other drugs.	Institutional collectivism and assertiveness at the country level was positively associated with desire not to have people who use alcohol or drugs as neighbors. Future orientation was negatively associated with desire not to have people who use alcohol as neighbors. Assertiveness was negatively associated with desire not to have people who use drugs as neighbors.	Structural stigma Passive observational
Rushovich et al. (2022)^70^ United States	Index—area characteristics	An index of economic hardship was calculated using American Community Survey 5-year population estimates for each census tract in Chicago. The index consisted of six indicators: unemployment, dependency (percent of population under age 18 or older than age 64), education, per capita income, crowded housing, and poverty.	Black, Latino, and White decedents from opioid overdose in Chicago	Opioid-related overdose deaths as indicated on death certificates	Overdose rates among Black and White individuals were higher in areas with greater economic hardship.	Structural racism Passive observational
South et al. (2023)^71^ United States	Single indicator—area characteristics	Housing remediation in predominantly Black neighborhoods was measured as (1) full remediation (installing working windows and doors, cleaning trash, weeding); (2) trash cleanup and weeding only; and (3) a no-intervention control.	Predominantly Black persons in Philadelphia	Counts of incidents of illegal substance trafficking and use as well as public drunkenness based on Philadelphia Police Department databases	Using a difference-in-differences analysis, housing remediation was not found to be associated with incidents of illegal substance trafficking and use or incidents of public drunkenness.	Structural racism Quasi-experimental
**Mental Health Studies**						
**Outcome: Depression**						
Miedema et al. (2019)^41^ Multinational	Multiple single indicators—community opinions/norms and government legislation/policy	Norms at the census tract level (or reasonable proximity) were measured using the United Nations Multi-country Study on Men (*N =* 6,164). Norms were the proportion of all men in the census tract who believed that it was shameful to have a gay son, the proportion who believed that there should not be laws and policies to protect gay people, and the proportion who reported perpetration of male-on-male rape or sexual assault. Also measured was the presence of any anti-gay laws/policies in each of the four Asia-Pacific countries included in the study.	562 sexual minority men in four countries in the Asia-Pacific region	Depressive symptoms assessed using an abbreviated version of the CES-D	Lower depressive symptoms were reported by sexual minority men respondents who lived in census tracts that had higher proportions of persons who believed it was shameful to have a gay son. (The authors noted that this finding was contrary to their expectations.) The other structural measures did not have statistically significant relationships with depressive symptoms.	Structural stigma Passive observational
Polos et al. (2022)^48^ United States	Index—institutional policy/action	A school contextual disadvantage index (CDI) measured differences in schools regarding resources and opportunities that have been partly determined by socio-historic structural racism. A school structural racism index (SRI) measured differences in school resources and opportunities between Black and White students within schools. A mean for each index was assigned to each school in the study.	12,112 respondents to the National Longitudinal Survey of Adolescent to Adult Health who were linked with a school disadvantage index, and 8,020 who were linked with a school structural racism index	Depression assessed using the 5-item CES-D	Higher CDI levels were associated with depressive for CDI, SRI was associated with symptoms. When controlling depressive symptoms among Black boys and girls but not among White boys and girls.	Structural stigma Passive observational
Pachankis et al. (2021)^43^ Multinational	Composite index—government legislation/policy with other subjects	Composite index consisting of country-level measure of 15 laws and policies related to sexual orientation (e.g., public accommodations protections) combined with country-level attitudes toward sexual minorities	Respondents to the European Men-Who-Have-Sex-With-Men internet survey who lived in their country of birth (178 countries, 106,883 respondents) or who moved from higher to lower structural stigma countries (48 countries, 11,831 respondents)	Depression assessed using the Patient Health Questionnaire-2	Among respondents still in their country of birth, structural stigma was related to depression. Among those who moved, longer exposure to the lower structural stigma environments of their receiving countries was associated with lower risk of depression. The association between country-level structural stigma and depression was partly mediated by internalized gay-negativity and social isolation.	Structural stigma Passive observational
Ünsal et al. (2023)^54^ Multinational	Composite index—government legislation/ policy with other subjects	Composite index consisting of (1) a sexual orientation index (countries were scored according to their sexual orientation-related protective laws and policies), (2) a gender orientation index (countries were scored according to their gender identity-related laws and policies), and (3) a measure of attitudes toward sexual and gender minority (SGM) individuals (responses to a question about level of comfort with having “a gay, lesbian, or bisexual person” or “a transgender person” in the highest elected political position in the country)	117,760 adults in 28 European Union member and candidate states (62,939 sexual minority men, 38,976 sexual minority women, and 15,845 gender minority adults)	Depression was assessed with a single question: “Have you been feeling downhearted or depressed over the last 2 weeks?”	For sexual minority men and women, structural stigma moderated how sexual or gender identity disclosure mediated the association between community participation (measured as participation in organizations for SGM people) and depression. For sexual minority men, structural stigma moderated how victimization (measured as frequency of physical or sexual attacks due to sexual or gender identity) mediated the association between community participation and depression.	Structural stigma Passive observational
Runkle et al. (2022)^69^ United States	Multiple single indicators—area characteristics	Green space per county, area of available green space per person in each county, percentage of total county population that lived within a 10-minute walk to green space; each measure was divided into tertiles	Hospital delivery discharges for pregnant women in South Carolina (*N=*238,922)	Major depressive disorder and mental disorders of pregnancy assessed using patient ICD-9 codes	Women in the lowest tertile for green space per county had higher risk for depression and pregnancy-related mental disorders. Those in the lowest two tertiles for available green space per person also were at higher risk for depression and pregnancy-related mental disorders. Women residing in counties in the middle tertile for the 10-minute walk to green space measure had higher risk for depression than those in the top tertile (the tertile with the greatest 10-minute walk access). Effects were generally more pronounced for Black women.	Structural racism Passive observational
Thyden et al. (2023)^72^ United States	Single indicator—institutional policy/action	Attendance at historically Black colleges or universities (HBCUs) or predominantly White institutions (PWIs)	488 Black students who had attended HBCUs or PWIs	Depression assessed using the CES-D	No overall association was found between depressive symptoms and attending HBCUs vs. PWIs. However, among those who attended high school outside of the southern United States, HBCU attendance appeared to have a protective association with depressive symptoms 7 years later.	Structural racism Passive observational
Martz et al. (2021)^65^ United States	Index—area characteristics	Latent profile analysis was used to identify four neighborhood typologies: Highly Segregated/Low-Socioeconomic Status (SES), Highly Segregated/Mid-SES, Moderately Segregated/ Mid-SES, and Integrated/ High-SES. The latent profile analysis used census tract data from the Atlanta, GA, MSA: percent poverty, percent unemployment, percent on supplemental Nutritional Assistance, percent with high school degree or less, and percent of Black individuals. The difference between Black-White composition at the census-tract level vs. the broader Atlanta, GA, MSA also was used.	428 women in Atlanta, GA, who were in the Black Women’s Experience Living with Lupus study in 257 census tracts	Self-reported depression	A direct association between segregated census tracts and depression was not indicated. Highly segregated census tracts were associated with higher levels of depression via increased subjective assessments of neighborhood disorder.	Structural racism Passive observational
Das et al. (2021)^58^ United States	Single indicator—law enforcement	Police killings of unarmed Black persons, as indicated in the Mapping Police Violence database	Depression-related emergency department visits among Black persons in 75 metropolitan counties during a 3-year period	Emergency department visits related to depression	Police killings of unarmed Black persons corresponded with an 11% increase in emergency department visits per 100,000 population related to depression among Black persons in the concurrent month and 3 months following the killings.	Structural racism Passive observational (ecological correlation)
**Outcome: Depression and Anxiety**						
Horne et al. (2021)^39^ United States	Single indicator—Government legislation/policy	Failed state (Massachusetts) referendum to invalidate extant protections based on gender	LGBTQ participants (*N =* 117)	Depression assessed using the CES-D-10 and anxiety assessed using the GAD-7	Depression and anxiety symptoms were lower following failure of the referendum (i.e., lower post-failure compared to pre-failure).	Structural stigma Quasi-experimental
Rucco et al. (2023)^50^ Italy	Single indicator—Government legislation/policy	Introducing, and then not passing, a bill titled “Measures to prevent and combat discrimination and violence on grounds of sex, gender, sexual orientation, gender identity, and disability.”	Sample of 381 bisexual+ respondents before the bill was rejected. Different sample of 299 bisexual+ respondents after the bill was rejected.	Depression assessed using the CES-D and anxiety assessed using the 21-item Beck Anxiety Inventory	Depression and anxiety were higher in the group surveyed after the bill failed to pass.	Structural stigma Quasi-experimental
Galán et al. (2022)^61^ United States	Index—historical trauma	Black parents’ experiences of racial discrimination measured using the 9-item Microaggression Scale	252 Black parent-child dyads in Pittsburgh, Pennsylvania	Youth self-reported depressive and anxiety symptoms	Parents’ experiences of racial discrimination were linked with higher levels of parent-child conflict, which in turn predicted youth-reported depression.	Structural racism Passive observational
Njoroge et al. (2022)^67^ United States	Index—area characteristics	Neighborhood SES index was created using census-based geocoding of neighborhood variables (e.g., percent of residents in poverty, percent married, percent with < high school education, median family income) linked to participant zip code. A Home Owners’ Loan Corporation-informed redlining measure also was examined.	Black birthing individuals from prenatal clinics in an urban university medical center (*N =* 151)	Depression assessed using the Edinburgh Postnatal Depression Scale and anxiety assessed using the GAD-7	Neighborhood socioeconomic status was not found to be associated with depression and anxiety. Redlining (i.e., the systematic implementation of discriminatory lending practices that denied mortgages in neighborhoods of color) was associated with depression, but not anxiety.	Structural racism Passive observational
**Outcome: Anxiety/Stress/Distress**						
Passell et al. (2022)^45^ Multinational	Composite index—government legislation/policy with other subjects	Marriage inequality (lack of legal recognition of same-sex marriages or civil unions) and criminalization of same-sex acts within individual countries as documented in the report State-Sponsored Homophobia from the Lesbian, Gay, Bisexual, Trans, and Intersex Association. The two measures were combined.	Internet survey respondents, including sexual minority respondents, (*N =* 5,929) from 13+ countries	Anxiety assessed using the GAD-7	Anxiety symptoms were not found to be associated with an interaction between social orientation and the marriage inequality/criminalization of same-sex acts index.	Structural stigma Passive observational
Frost (2020)^34^ United Kingdom	Single indicator—community opinions/norms	Percentage of “leave” voters (i.e., voters in favor of Brexit) in respondent’s place of residence	Adult migrants (*N =* 311) living in the United Kingdom	Anxiety assessed using the GAD-7	Migrants living in areas with higher percentages of “leave” voters had more experiences of discrimination (self-reported), which in turn was associated with more anxiety.	Structural stigma Passive observational
Woodford et al. (2018)^56^ United States	Multiple single indicators—institutional policy/action	Based on interviews with staff at colleges and on the colleges’ website information, the existence of 11 policies and resource programs designed to promote the inclusion and well-being of sexual and gender minorities (LGBTQ) was determined: gender-identity-inclusive antidiscrimination, domestic partner benefits, name of choice, transition coverage, LGBTQ office/staff, general LGBTQ campus awareness, LGBTQ ally/safe space, LGBTQ student support initiative, for-credit LGBTQ course, LGBTQ alumni initiative, ratio of LGBTQ student organizations to the student population.	268 cisgender LGBQ+ students at 58 colleges	Anxiety assessed using the GAD-7 and stress assessed using the 10-item Perceived Stress Scale	Antidiscrimination policies inclusive of gender identity and sexual orientation, offering at least 1 for-credit LGBTQ course, and a higher ratio of LGBTQ student organizations to the student population were directly associated with participants reporting lower levels of discrimination. In turn, lower levels of discrimination were associated with less psychological distress (measured by the perceived stress and anxiety scales).	Structural stigma Passive observational
Hatzenbuehler et al. (2018)^36^ Sweden	Composite index—government legislation/policy with other subjects	Composite index consisting of a measure of Swedish laws and policies toward sexual minorities combined with a measure of social attitudes from the European Social Survey (whether “gays and lesbians should be free to live their own life as they wish”) measured at multiple points in time.	Sweden: gay, lesbian, bisexual, and heterosexual participants in national surveys at three points in time separated by 5-year intervals (*N =* 23,248) from 2010 to 2015	Psychological stress as measured by the 12-item General Health Questionnaire	Structural stigma decreased over time, and this was associated with lower levels of psychological distress among gay men and lesbians but not among bisexuals and heterosexuals. In 2015, a sexual orientation disparity (gay/lesbian vs. heterosexual) in psychological distress was no longer indicated.	Structural stigma Passive observational
Do et al. (2019)^59^ United States	Index—area characteristics	D-index and P-index (measures of metropolitan segregation); neighborhood poverty measure constructed using the federal poverty level in census tracts	Black and White respondents to the American Community Survey	Kessler 6 scale, a measure of non-specific psychological distress	Higher D- and P-index measures were associated with higher odds of psychological distress for Black respondents. Neighborhood poverty explained some but not all of the association.	Structural racism Passive observational
**Outcome: Eating Disorders**						
Hoggard et al. (2023)^62^ United States	Index—area characteristics	Neighborhood disadvantage index derived from the American Community Survey. It was the average of five census indicators: proportion non-Hispanic Black, proportion of female headed families with children, proportion of households with public assistance income or food stamps, proportion of families with income below the federal poverty level, and proportion of population age 16 and older that was unemployed.	751 Black participants (ages 18 to 88) recruited using Qualtrics Panels, an online survey service	Emotional eating assessed using the Emotional Eating Scale, a 25-item self-report measure	Neighborhood disadvantage was not found to be associated with emotional eating.	Structural racism Passive observational
Beccia et al. (2022)^73^ United States	Composite index—government legislation/policy with other subjects	Composite index of political participation, state policies, socioeconomic measures, institutional support, and access to reproductive care indicating high or low levels of structural sexism. Number of years in a high structural sexism state	Cohort of cisgender participants who were aged 9 to 14 in 1996 and were followed through 2016 in the Growing Up Today Study (*N =* 16,875).	Chronic dieting, purging, binge eating, overeating	Each additional year of living was associated with a 5% increased risk of purging, an 8% increased risk of binge eating, and a 9% increased risk of overeating. Risks on average were greater for girls/women than for boys/men.	Structural sexism Quasi-experimental
**Outcome: General Mental Health**						
Perales and Todd (2018)^47^ Australia	Single indicator—community opinions/norms	Using data from a national postal plebiscite on same-sex marriage legislation (the Australian Marriage Law Postal Survey), the percent of voters who voted “No” within each of 135 electorates was determined and linked to individual respondents in the Household, Income, and Labor Dynamics in Australia Survey.	LGB and general population participants (*N =* ~15,000) in the Household, Income, and Labor Dynamics in Australia Survey	Mental health assessed using the mental health subscale of the 36-Item Short Form Survey (SF-36), known as the Mental Health Inventory or MH-5	Compared to respondents who did not self-identify as LGB, LGB respondents reported worse mental health in electorates with higher percentages of “No” voters.	Structural stigma Passive observational
van der Star et al. (2021)^55^ Multinational	Composite index—government legislation/policy with other subjects	Composite index consisting of (1) attitudes within countries towards sexual minorities obtained from the Global Acceptance Index and (2) data on discriminatory laws and unequal policies obtained from a report by the International Lesbian, Gay, Bisexual, Trans, and Intersex Association	247 non-heterosexual men who resided in Sweden but were born in another country	Mental health problems assessed using the Symptom Inventory-18 scale	Country-of-origin structural stigma was associated with poor mental health, and this association was mediated by the participants’ rejection sensitivity and internalized homophobia, but only among those who arrived in Sweden at an older age and more recently.	Structural stigma Passive observational
Duncan et al. (2023)^60^ United States	Multiple single indicators—community opinions/norms	Socio-spatial self-organizing maps that used data from Twitter to create contiguous, non-overlapping “clusters” (i.e., neighborhoods) characterized by consistent levels of racism and homophobia	147 young sexual minority men in New York City	Participants reported how many days their mental health was not good (e.g., the number of days they felt stressed or depressed) during the preceding 30 days.	Spending more time in more racist spaces was associated with more days of poor mental health. This association was greater for White and Black young sexual minority men than for Latino counterparts. More time in more homophobic spaces was not found to be associated with days of poor mental health.	Structural racism Passive observational
Hswen et al. (2020)^63^ United States	Single indicator—community opinions/norms	Time series of percent of tweets regarding Mexicans and Hispanics that were negative	8,314 Hispanic Gallup poll respondents	Time series of negative mental well-being measured as daily worry	Negative percent of tweets was associated with reports of negative mental well-being.	Structural racism Passive observational
Lynch et al. (2021)^64^ United States	Index—access to financial loans	Combination of two lending measures: (1) historic redlining as indicated by Home Owners’ Loan Corporation grades and (2) current lending discrimination (i.e., census tracts with low lending occurrence and/or high-cost loans)	Census tracts in Milwaukee, Wisconsin	Self-reports of poor mental health for ≥ 14 days for City of Milwaukee census tracts, obtained from the 500 Cities Project	Historic redlining and current lending discrimination were associated with higher self-reports of poor mental health.	Structural racism Passive observational (ecological correlation)
Nelson et al. (2023)^42^ United States	Index—government legislation/policy	Data from the Movement Advancement Project’s Equality Maps, which tallied the number of LGBT-related laws and policies. Laws and policies that were protective for LGBT people were coded 1, those that were harmful were coded -1.	10,032 sexual minority and 1,072 transgender BRFSS respondents ages ≥ 50 years	Number of days when mental health was not good	Gay men living in states with fewer LGBT protections had higher odds of reporting higher numbers of days with poor mental health.	Structural stigma Quasi-experimental
**Outcome: Mental Health Services**						
Paula et al. (2014)^46^ Brazil	Single indicator—regional development	Residence in less-developed vs. more-developed regions of Brazil	Survey of 6- to 16-year-olds (*N =* 1,721) in four regions of Brazil	Use of mental health services in the previous 12 months	Children in less developed (more deprived) regions had lower rates of mental health service use, even when the presence of psychiatric disorders was taken into account.	Structural stigma Passive observational
Roulston et al. (2022)^49^ United States	Index—community opinions/norms	State-level anti-Black racism was measured using factor analyses of publicly available data from Project Implicit. A state-level homophobia measure was similarly constructed. A mental health care provider shortage index obtained from the Health Resources and Services Administration was used. The Gini coefficient (a measure of income inequality) for each state was used.	Adolescents from 43 states who self-identified as sexual minority youth of color and who endorsed a desire for mental health support during the COVID-19 pandemic (*N =* 470)	Self-reports of never, sometimes, or always accessing mental health support during the COVID-19 pandemic	Health care provider shortage estimates were associated with reports of having never (vs. always) accessed mental health support during the pandemic. Respondents in areas with both lower homophobia and lower anti-Black racism were more likely to report always (vs. sometimes) accessing mental health treatment.	Structural stigma Passive observational
WHO/Demyttenaere et al. (2004)^30^ Multinational	Composite index—economic/social/vulnerability dimensions	Developed countries vs. less-developed countries as indicated by a composite index consisting of economic measures, social measures, and vulnerability	Persons with DSM-IV disorders among 60,463 community adult respondents in 14 countries	Treatment for serious mental health disorders	35.5% to 50.3% of serious cases in developed countries and 76.3% to 85.4% in less developed countries received no treatment in the 12 previous months.	Structural stigma Passive observational
Bennewith et al. (2010)^57^ England	Single indicator—institutional policy/action	Involuntary psychiatric hospitalization of patients	102 Black, 33 Asian, 18 mixed ethnicity, and 392 White patients at 22 hospitals managed by mental health trusts in London and in the southeast, northwest, and southwest of England	Hospital records showing forced medication, use of restraints, and seclusion	No statistically significant differences in coercion by ethnicity of patient were found.	Structural racism Passive observational
**Outcome: Mental Health Legislation**						
Conley (2021)^28^ United States	Index—government legislation/policy	Legislative mental health bills were coded as having stigmatic language if three or more of the following were present: labeling, stereotyping, separating (us vs. them), status loss, and discrimination. An index of a bill’s potential effect was constructed by noting whether the bill reduced personal liberties, protections against discrimination, privacy, and resources/services. Bills with any of these items were coded as having stigmatic potential.	Sample of 200 mental health-related bills from 50 U.S. states	Passage of bill	Bills with stigmatic language were more likely to be passed. Stigmatic potential of a bill was not found to be associated with its passage.	Structural stigma Passive observational
Conley and Baum (2023)^29^ United States	Multiple single indicators—government legislation/policy	U.S. political party (Republican or Democrat) of a mental health-related bill’s sponsor, the majority gender of the bill’s political sponsor(s), whether political party of the bill’s sponsors was in the minority or majority of the chamber (House/Senate) of the bill’s introduction	Sample of 200 mental health-related bills from 50 U.S. states (January 2017 through October 2019)	Introduction of bills with stigmatic language or bills with potential effect regarding reduced personal liberties, protections against discrimination, privacy, and resources/services	Republican sponsors were more likely to introduce bills with stigmatizing language. No associations with potential effect of bills were found.	Structural stigma Passive observational
**Substance Use and Mental Health Studies**						
**Outcome: Substance Use and Mental Health**						
Everett et al. (2016)^32^ United States	Single indicator—government legislation/policy	Before Illinois civil union bill was signed, after the bill was signed but before it was enacted (January 31, 2011–May 31, 2011), and after the bill was enacted	517 English-speaking Black, Latina, and White women who self-identified as lesbian; unspecified numbers of persons in this group were surveyed during each of the three time periods noted in the description of the structural measure	Self-reported heavy episodic drinking (ever had 6+ drinks in a day during past 12 months), intoxication, alcohol dependency, adverse drinking consequences; depressive symptoms assessed using the CES-D-10	Black and Latina women interviewed after the bill was signed reported fewer depressive symptoms than those interviewed before the bill was signed. After the bill was enacted, heavy episodic drinking reported by the full study sample was higher.	Structural stigma Quasi-experimental
Cascalheira et al. (2022)^27^ United States	Index—government legislation/policy	Movement Advancement Project index tracking state legislation relevant to the rights of SGM people. The score for each year was transformed. Also, to measure government funding, the number of substance use treatment facilities receiving such funding was aggregated into yearly state-level counts and transformed into yearly percentages.	Mental health and substance use treatment programs offering SGM-tailored programming	Number of facilities offering SGM-tailored programming	Legislation supportive of SGM civil rights was associated with increases in SGM-tailored programs at substance use facilities. Legislation was not found to be associated with SGM-tailored programming at mental health treatment facilities. No association with government funding was found.	Structural stigma Passive observational
Nardone et al. (2020)^66^ United States	Single indicator—access to financial loans	Historic redlining of census tracts in nine large cities as indicated by Home Owners’ Loan Corporation grades	8,122 census tracts	Self-reports of binge drinking, current smoking, and poor mental health at the census tract level obtained from the 500 Cities Project dataset	In several cities, census tracts with lower lending ratings were associated with higher estimates of poor mental health and current smoking but lesser binge drinking.	Structural racism Passive observational (ecological correlation)

* If a study used at least one index and no composite indexes, it was listed as having used an index, regardless of whether it also used single indicators.

*Note:* BRFSS, Behavioral Risk Factor Surveillance Survey; CDI, contextual disadvantage index; CES-D, Center for Epidemiologic Studies Depression Scale; COVID-19, coronavirus disease 2019; D-index, dissimilarity index; *DSM-IV, Diagnostic and Statistical Manual of Mental Disorders*, fourth edition; GAD-7, generalized anxiety disorder 7-item scale; HBCUs, historically Black colleges or universities; ICD-9, *International Classification of Diseases, 9th Revision;* LGB, lesbian, gay, or bisexual; LGBQ, lesbian, gay, bisexual, or queer; LGBT, lesbian, gay, bisexual, or transgender; LGBTQ, lesbian, gay, bisexual, transgender, or queer; MSA, metropolitan statistical area; PWIs, predominantly White institutions; P-index, proportional index; SES, socioeconomic status; SGM, sexual and gender minority; SRI, structural racism index; VHA, Veterans Health Administration; WHO, World Health Organization
